# Evaluating sheep hemoglobins with MD simulations as an animal model for sickle cell disease

**DOI:** 10.1038/s41598-023-50707-y

**Published:** 2024-01-02

**Authors:** Caroline E. Kuczynski, Christopher D. Porada, Anthony Atala, Samuel S. Cho, Graça Almeida-Porada

**Affiliations:** 1 Wake Forest Institute for Regenerative Medicine, Winston-Salem, NC 27101 USA; 2 Department of Physics, Wake Forest University, Winston-Salem, NC 27109 USA; 3 Department of Computer Science, Wake Forest University, Winston-Salem, NC 27109 USA

**Keywords:** Computational biophysics, Molecular conformation, Protein analysis, Sickle cell disease, Mathematics and computing

## Abstract

Sickle cell disease (SCD) affects millions worldwide, yet there are few therapeutic options. To develop effective treatments, preclinical models that recapitulate human physiology and SCD pathophysiology are needed. SCD arises from a single Glu-to-Val substitution at position 6 in the β subunit of hemoglobin (Hb), promoting Hb polymerization and subsequent disease. Sheep share important physiological and developmental characteristics with humans, including the same developmental pattern of fetal to adult Hb switching. Herein, we investigated whether introducing the SCD mutation into the sheep β-globin locus would recapitulate SCD’s complex pathophysiology by generating high quality SWISS-MODEL sheep Hb structures and performing MD simulations of normal/sickle human (huHbA/huHbS) and sheep (shHbB/shHbS) Hb, establishing how accurately shHbS mimics huHbS behavior. shHbS, like huHbS, remained stable with low RMSD, while huHbA and shHbB had higher and fluctuating RMSD. shHbB and shHbS also behaved identically to huHbA and huHbS with respect to β_2_-Glu6 and β_1_-Asp73 (β_1_-Asn72 in sheep) solvent interactions. These data demonstrate that introducing the single SCD-causing Glu-to-Val substitution into sheep β-globin causes alterations consistent with the Hb polymerization that drives RBC sickling, supporting the development of a SCD sheep model to pave the way for alternative cures for this debilitating, globally impactful disease.

## Introduction

Sickle cell disease (SCD) is the most common inherited blood disorder, affecting ~ 100,000 patients in the US and millions more worldwide^1,2^. The World Health Organization (WHO) officially recognized SCD as a global problem with significant economic implications and has made the dismal forecast that the incidence of SCD will rise to > 400,000 births/year by 2050^3,4^. SCD is caused by a single nucleotide substitution that results in the replacement of the glutamic acid at position 6 with valine of the β-subunit (β-Glu6Val) of hemoglobin (Hb), leading to polymerization of the β-globin chains of the abnormal sickle hemoglobin (HbS) when in the deoxygenated T-state, the formation of rigid, insoluble HbS fibers, and subsequent red blood cell (RBC) sickling^5–10^. This seemingly extraordinarily minor genetic perturbation, dubbed “a molecular disease” by Pauling et al.^11^, leads to hemolysis, inflammation, vaso-occlusion, and vasculopathy, collectively producing extreme pain and a myriad of acute and chronic complications, requiring frequent emergency room visits and hospitalizations^12^.

Gene therapy is actively being explored [ClinicalTrials.gov ID# NCT02140554] as a treatment for SCD, but the ongoing trials are still too early to draw definitive conclusions as to long-term efficacy and safety. To-date, four disease-modifying pharmaceuticals have been approved by the FDA. However, these agents must be administered throughout life on a highly regimented schedule, they are not effective in all patients, and they have numerous side effects that reduce patient adherence and often preclude patients from continuing therapy^13,14^. At present, the only curative treatment available for SCD is hematopoietic stem cell (HSC) transplantation. However, as currently performed in the postnatal setting, this procedure poses considerable challenges, especially when the patient is from an ethnic group that is underrepresented in the HSC donor bank, and it carries the risk of serious morbidities, and even mortality^14^. There is an urgent need to develop novel approaches to treating SCD that are safe and effective for all patients.

During development, the fetus is protected from sickling by the presence of fetal hemoglobin (HbF), but as HbF levels decline after birth, the hemolytic anemia and associated chronic organ damage of SCD, as well as other clinical complications such as stroke, splenic crisis, pain episodes, life-threatening infections, and episodes of acute chest syndrome, can begin as early as 6 months of age. This early onset of clinical symptoms and complications has prompted the recent recommendation that the standard of care for all infants with SCD should be to commence disease-modifying therapy, such as hydroxyurea, as early as possible^15^. SCD would thus be an ideal disease to correct neonatally, or even prenatally, if sufficient levels of engraftment of healthy donor or autologous gene-corrected HSC could be achieved. For such treatments to become a reality, however, preclinical animal models that accurately recapitulate human development, physiology, hematopoiesis, and SCD pathophysiology are urgently needed^16^.

While mice have been a cornerstone of SCD research for decades, mice, unlike humans, lack HbF and undergo only a single switch from embryonic to adult Hb during development^17^. As such, they cannot be used to study agents, such as hydroxyurea, that exert their therapeutic effect by reactivating expression of HbF. This critical developmental difference also limits their utility for exploring prenatal approaches to treating SCD. While transgenic mice harboring portions of the human globin gene locus have been created to overcome this limitation^17,18^, the creation of an SCD model in a large animal that harbors an endogenous globin locus that mirrors that of humans, and would enable long-term evaluation of the chronic aspects of SCD and the efficacy of new therapeutic approaches, is highly desirable. Among large animals, sheep (*Ovis aries*) share many important physiological and developmental characteristics with humans, and have, therefore, been used extensively in the study of mammalian fetal physiology, and results obtained with this model have been directly applicable to the understanding of human fetal growth and development. Importantly, sheep are close in size to humans during development and at birth, their immune development parallels that of humans; like humans, sheep naturally undergo changes in the primary sites of hematopoiesis during gestation. Moreover, as a large, long-lived animal (lifespan 8–12 years), sheep allow critical questions of long-term efficacy and safety to be properly addressed. Over the past several decades, these features have led investigators to use sheep as a highly translationally relevant animal model for a variety of hematologic and obstetric diseases^19–22^. Of direct relevance to SCD, unlike mice, sheep exhibit the same developmental pattern of fetal to adult hemoglobin switching as humans^23,24^. In addition, BLAST pairwise alignment of the DNA coding sequences (CDS) of the HBB gene, which encodes β-globin (using RefSeq ID NM_000518.5 and GenBank ID DQ352471.1, respectively)^25–28^, demonstrated ~ 86% sequence identity exists between the normal deoxygenated adult human Hb (huHbA) and normal deoxygenated adult sheep Hb (shHbB) at the nucleotide level. For these reasons, we sought to determine whether introducing the human SCD mutation into the HBB locus in sheep to create the mutant “shHbS” would better recapitulate the complex physiology of SCD in human patients than current models, creating a platform that enables straightforward translation to humans^16,29,30^ for future development of cell and gene therapies, and paving the way for the development of alternative cures for this debilitating, globally impactful disease.

**Figure 1:**
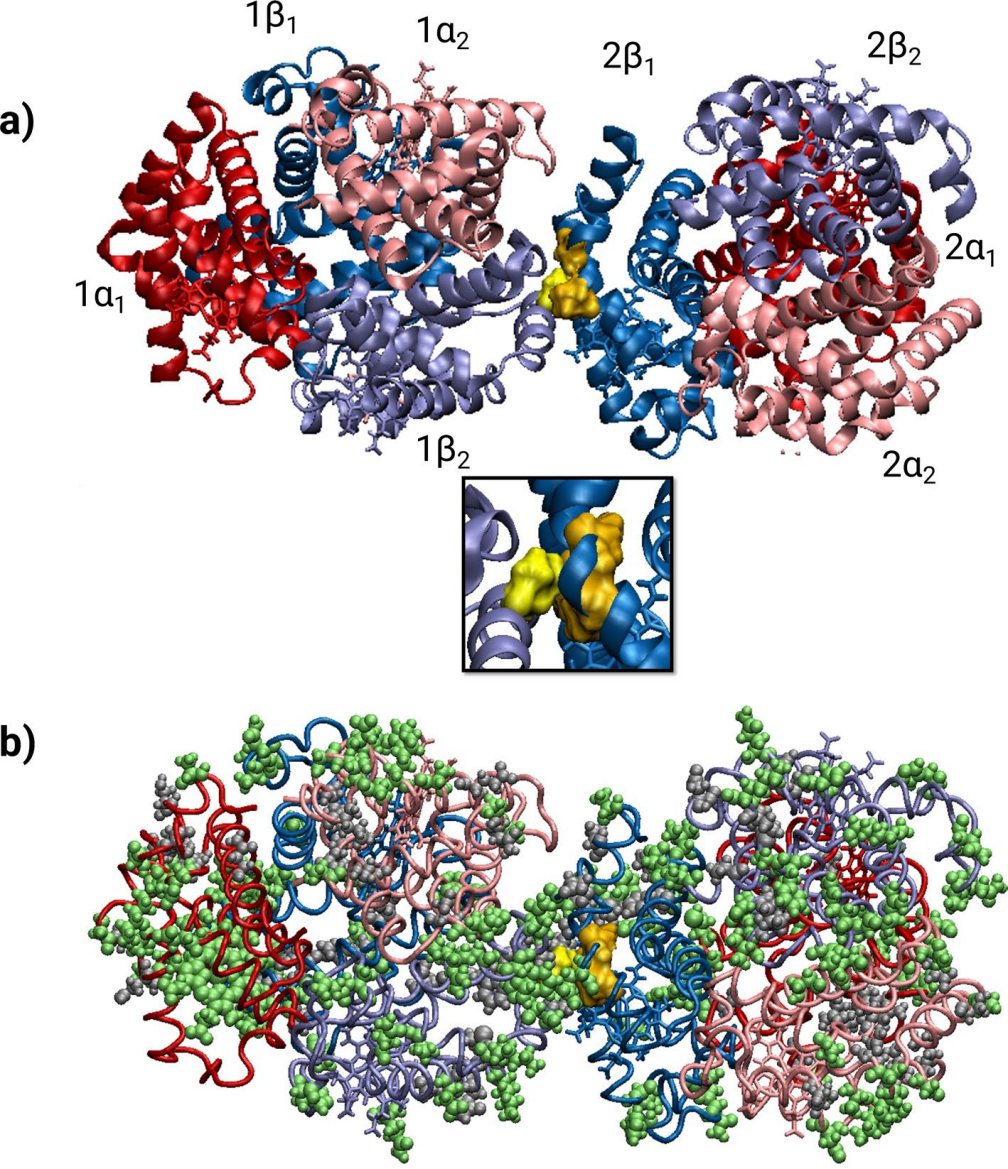
Structures of sickle hemoglobin from human (huHbS) and sheep (shHbS). (**a**) The X-ray crystallographic structure of huHbS as a dimer of two sickle Hb tetramers (PDB: 2HBS). The α-subunits are in red and pink, and the β-subunits are in blue and light blue. The proteins are shown in a ribbon representation and the hemes are in a stick representation. The 1β2-Val6 is shown in light yellow, and the hydrophobic pocket formed by residues 2β1-Ala70, 2β1-Phe85, and 2β1-Leu88 is shown in dark yellow in a space-filled representation. Below it is a close-up view of the interface of interaction with the hydrophobic pocket that is created by the 1β_2_-Val6 mutation. (**b**) The SWISS-MODEL modeled shHbS. The residues that are different between huHbS and shHbS are represented in a space-filled representation, with dissimilar residues colored green and similar residues colored grey. The shHbS α- and β-subunits are in a tube representation and colored the same as in (**a**), and the 1β2-Val5 and the hydrophobic pocket residues (2β1-Ala71, 2β1-Phe84, and 2β1-Leu87) are also represented the same. As detailed in the Materials and Methods, images were created by the authors using Visual Molecular Dynamics (VMD) software, v. 1.9.4: https://www.ks.uiuc.edu/Research/vmd/.

Prior to undertaking the creation of this new SCD model, we herein conducted molecular dynamics (MD) simulations with a modeled structure of the mutant shHbS to characterize the interactions between the β-globin chains and compare them to huHbS to predict if the introduction of the SCD mutation into the sheep HBB gene would result in Hb polymerization and RBC sickling in vivo. Since the structure of huHbA was first resolved via X-ray crystallography^31^, decades have been dedicated to the elucidation of its structure and function, via both in vitro and in silico methods; huHbA is now a classical model for fundamental processes such as allostery, cooperativity, and electron transfer mechanisms^32–34^. Each Hb molecule exists in RBCs as a tetramer consisting of a pair of α-subunits (α1 and α2) and a pair of β-subunits (β1 and β2), and each subunit has an obligatory iron-containing heme^35^ that carries oxygen from the lungs to the rest of the body (Fig. 1a). During this process, Hb undergoes a conformational change from the oxygenated R-state to the deoxygenated T-state. Over the relatively lengthy history of the structural study of Hb, understanding of its allosteric transition has evolved considerably over time; recent multiscale simulations have shown that both quaternary *and* tertiary structure modulate oxygen affinity, with proximal effects regulating affinity in the α chains and largely driving the allosteric transition, and a complex interplay of proximal and distal effects work to modulate β chain affinity^36^. However, unlike in huHbA, the β-Glu6Val substitution in SCD promotes inter-tetrameric Hb interactions through a lateral contact with a hydrophobic pocket comprised of β-Ala70, β-Phe85, and β-Leu88 in a second Hb molecule (Fig. 1a), which becomes surface-exposed by the R- to T-state conformational change, only then driving polymerization^37–39^. Hb polymerization is thought to occur through a double nucleation that begins with the formation of HbS fibers, followed by a nucleation of additional fibers on the surface of existing ones, resulting in RBC deformation and the subsequent deleterious effects of SCD. Recent studies, however, suggest that electrostatic interactions resulting from the loss of the negatively charged Glu significantly contribute to huHbS aggregation^40,41^.

**Figure 2:**
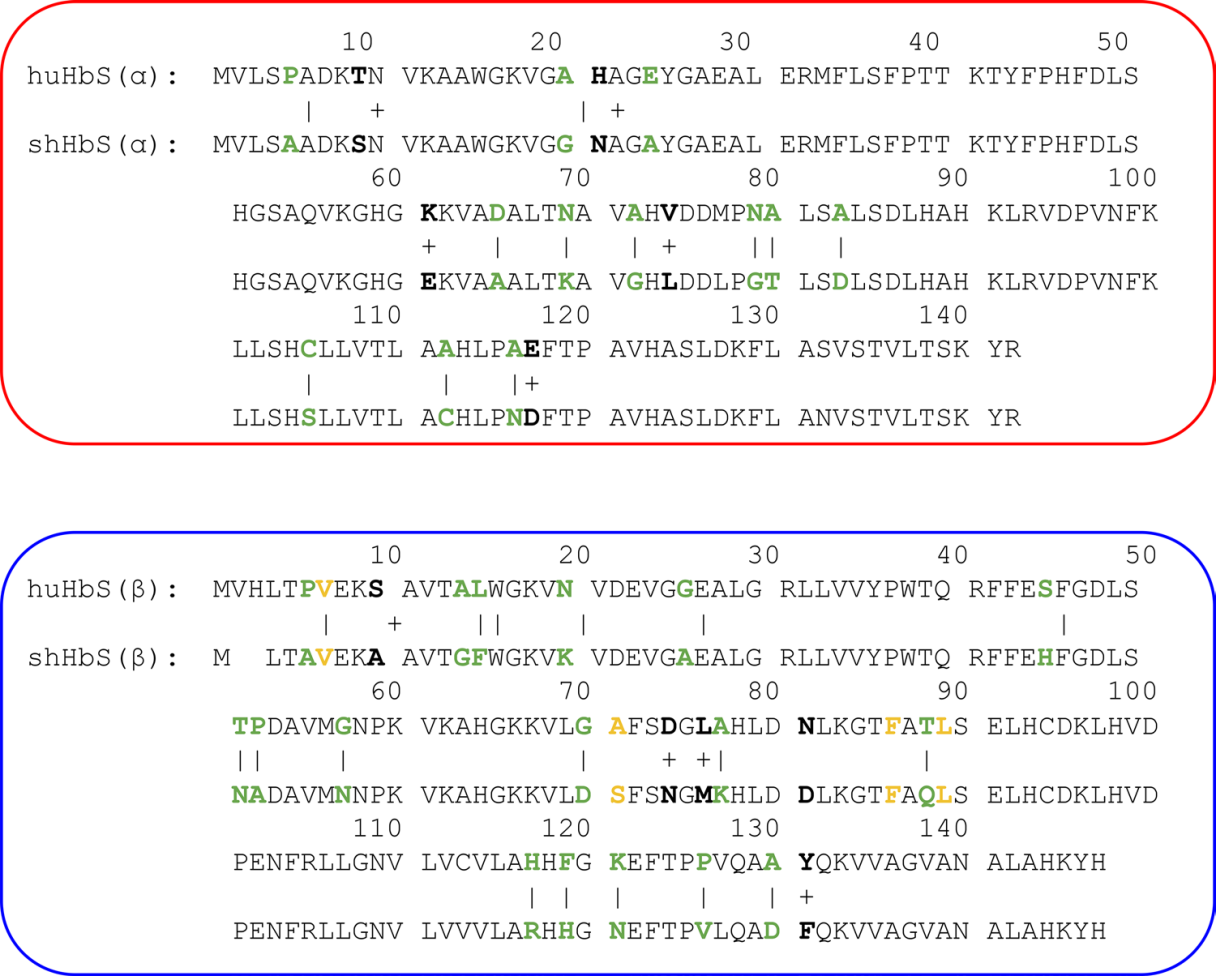
Amino acid sequences for huHbS and shHbS α- and β-subunits. The non-conservative substitutions are colored green, the conservative substitutions are colored bolded black, and the residues involved in the hydrophobic pocket are yellow. Substitutions are designated non-conservative or conservative according to BLAST output using the BLOSSUM62 matrix.

Unfortunately, while normal huHbA has been widely studied at the molecular, cellular, and physiological level for decades, rigorous studies of normal sheep hemoglobin (shHbB) are currently very limited, and molecular-resolution studies are currently absent in the field. Even with the high sequence identity between huHbA and shHbB, it is not immediately clear that huHbA and shHbB behave similarly at the molecular level due to numerous sequence differences, including amino acid insertions, deletions, and both conservative and non-conservative substitutions throughout the Hb molecule (Fig. 1b), including several amino acid substitutions in β-subunit residues adjacent to hydrophobic pocket residues (Figs. 1b, 2). We, therefore, employed MD simulations to model the shHbB molecule and to ascertain the impact that introducing the SCD-causing mutation would have on its behavior. MD simulations have proven to be an indispensable tool in structural biology for quantitatively characterizing the dynamics and protein–protein interactions for a plethora of proteins and biomolecular complexes^42–45^ including both normal huHbA and more atypical hemoglobins from other animals^40,41,46–51^. Since there exists a high-resolution structure of a dimer of two huHbS tetramers—thought to be the starting point for polymerization—we used this as a starting structure with SWISS-MODEL to model shHbS, along with normal huHbA and shHbB to serve as controls, for further study using MD simulations. We characterized the global stability, as well as the local residue-level pair distances between the Hb tetramers and the interactions with water in order to compare the human and sheep Hb in the normal and sickle forms.

## Results

### Long timescale MD simulations of huHbS and shHbS are globally stable

To quantitatively compare the human and sheep variants of the normal and sickle Hb, we performed 2 μs MD simulations of: (1) huHbA, (2) huHbS, (3) shHbB, and (4) shHbS. These MD simulations were carried out using the latest CHARMM36 force field with explicit waters using standard protocol and thus without kinetic biases. Only the huHbS is entirely empirically determined, and its two tetramer structure was used as a starting point to model the other structures. For the huHbA, the β-Val6 from huHbS was replaced with a Glu residue, while the shHbB and shHbS structures were modeled using SWISS-MODEL, as described in the preceding section and in the “Methods” section. In vivo, the deoxy-huHbA does not polymerize whereas the huHbS does.

**Figure 3:**
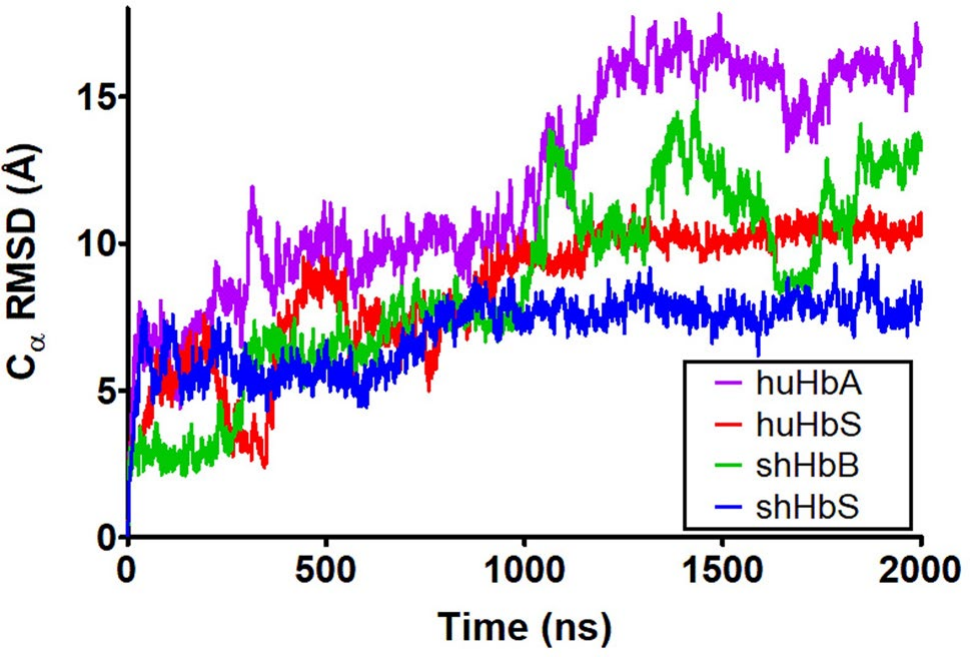
RMSD of the huHbA, huHbS, shHbB, and shHbS from 2 μs MD simulation trajectories. Global RMSD for the entire dimer of two Hb molecules.

**Figure 4:**
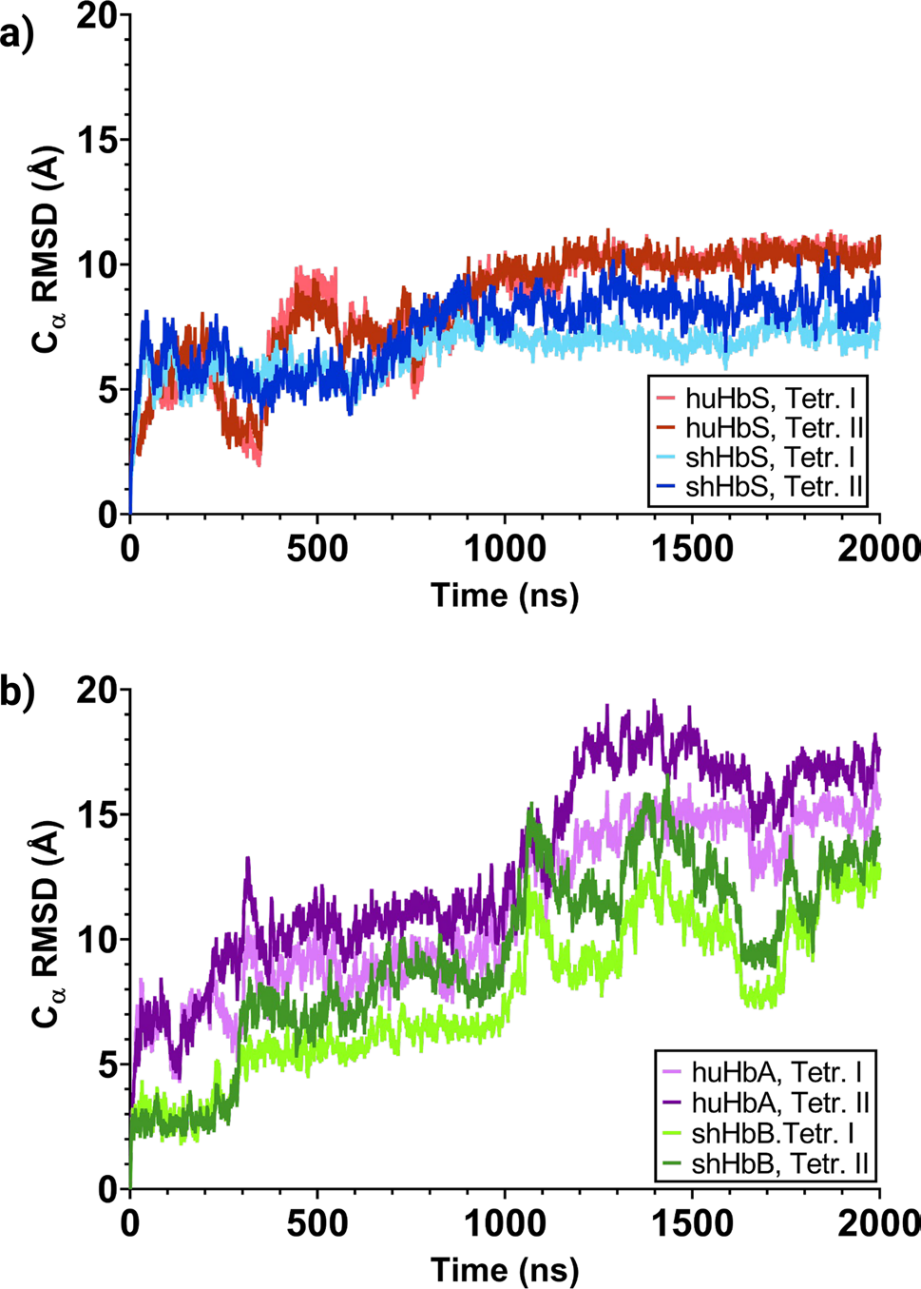
RMSDs per tetramer of (**a**) huHbS and shHbS, and (**b**) huHbA and shHbB, from 2 μs MD simulation trajectories. RMSDs shown here were calculated for individual tetramers with respect to their initial positions only.

In our 2 μs MD simulations (to our knowledge, the longest MD simulations for any Hb reported to date), the global RMSDs (Fig. 3) start at around 4 Å and steadily increase to about 7–10 Å for the first 1 μs. For the remaining 1 μs, the RMSDs for both the huHbS and shHbS remain steady in that range, while the RMSDs of the huHbA and shHbB increased to about 13–15 Å, and shHbB RMSD fluctuated over a broad range. Despite just the single nucleotide difference between the normal Hb and HbS, we observe significant differences by this global measure in both the human and sheep variants. Interestingly, even though the amino acid sequence identity between the huHbS and shHbS is only ~ 82%, they are similarly stable at this timescale while their normal counterparts, huHbA and shHbB, are not. Furthermore, when the RMSDs were calculated for individual tetramers over the entire trajectories, we observed similar patterns of relative stability of tetrameric RMSDs for huHbS and shHbS, and slightly increased, fluctuating tetrameric RMSDs for huHbA and shHbS (Fig. 4).

### Protein–protein interactions at the SCD mutation site are largely conserved between huHbS and shHbS

**Figure 5:**
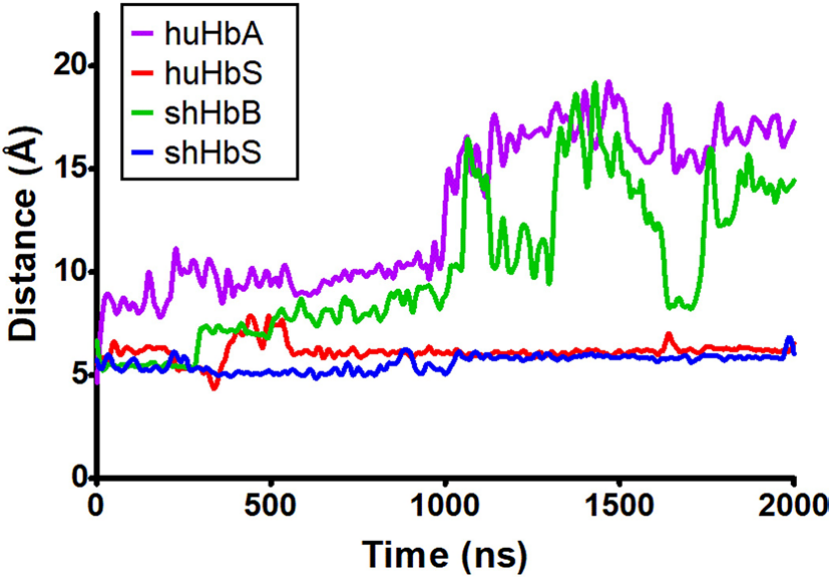
Center of mass distances between human 1β2-Glu6/Val6 or sheep 1β2-Glu5/Val5 and their respective hydrophobic pockets on chain 2β_1_ at the interaction interface between the two tetramers (as depicted in Fig. 1a inset) at the interaction interface between the two tetramers of the huHbA, huHbS, shHbB, and shHbS over 2 μs MD simulation trajectories.

We next explored the interactions between the human 1β2-Glu6/Val6 and sheep 1β2-Glu5/Val5 and their respective hydrophobic pockets by calculating the center of mass distance between them (Fig. 5). For both shHbS and huHbS, the center of mass distances remained around 6 Å. However, for huHbA and shHbB, their center of mass distance increased to about 6 Å in the first 1 μs of the trajectories, then increased to over 15 Å. This indicates that the Val in huHbS and shHbS similarly remains locked in place with the hydrophobic pocket for the duration of the MD simulation, while the Glu in huHbA and shHbB moves away from the hydrophobic pocket.

To further explore interactions between the aforementioned Glu/Val and the 2β1 chain on the adjacent Hb tetramer in each of the four different trajectories, we calculated the residue-residue pair center of mass distances between the Glu/Val and every other residue in the 2β1 chain for the last 1 μs of the simulations—after the RMSDs for huHbS and shHbS had stabilized—at intervals of 50 ns, and therefore 20 temporal sets of points per trajectory. We sought to observe if there was a similar pattern of residue-residue distances between the human 1ꞵ_2_-Val/Glu6 or sheep 1ꞵ_2_-Val/Glu5 and all residues in the 2ꞵ_1_ chain. We first standardized the data for each trajectory individually, using the equation z = (x − μ)/σ, where x is the raw data value, μ is the mean, and σ is the standard deviation. As a result, the mean value is 0 and each value represents its distance from the mean in units of standard deviation.

**Figure 6:**
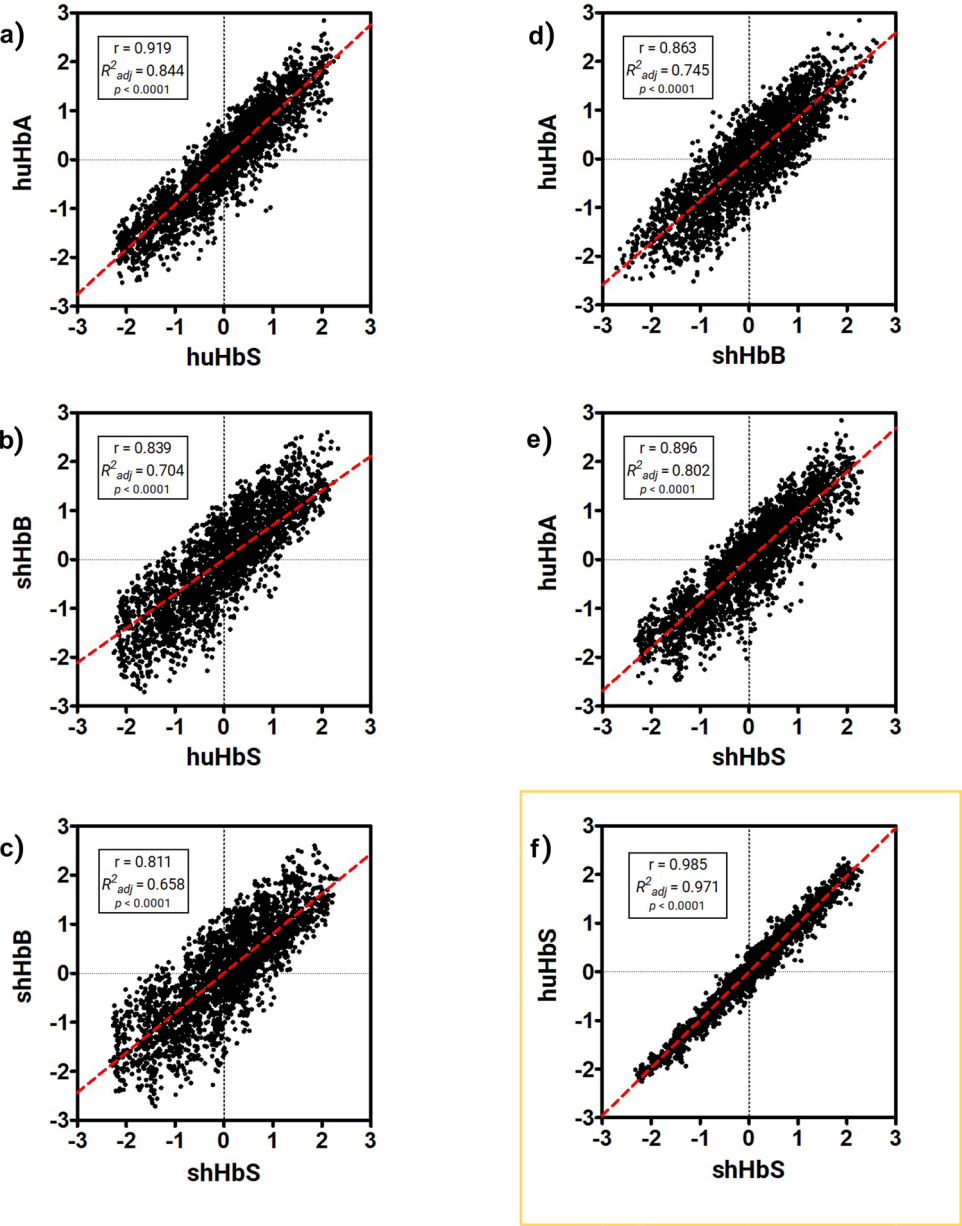
Correlation between center of mass distances between human 1ꞵ2-Val/Glu6 or sheep 1ꞵ2-Val/Glu5 and all other residues in chain 2ꞵ1, calculated from 1 to 2 μs, at every 50 ns, after standardization. Shown are the correlations between (**a**) HuHbA and HuHbS, (**b**) shHbB and HuHbS, (**c**) ShHbB and shHbS, (**d**) HuHbA and ShHbB, (**e**) HuHbA and shHbS, and (**f**) huHbS and shHbS, each with an inset consisting of the Pearson correlation coefficient (r), linear regression fitting (R2) and p-value (p).

After standardization, we calculated the Pearson correlation coefficient (r) and linear regression fitting (R2) for each pair of trajectories, for a total of six comparisons (Fig. 6). Notably, the highest r value was observed between huHbS and shHbS, at r = 0.985 (Fig. 6f). In contrast, the r values observed between huHbA and huHbS (r = 0.919) (Fig. 6a), shHbB and huHbS (r = 0.839) (Fig. 6b), shHbB and shHbS (r = 0.811) (Fig. 6c), huHbA and shHbB (r = 0.863) (Fig. 6d), and huHbA and shHbS (r = 0.896) (Fig. 6e), were of lesser magnitude. Taken together, these results show that the interface between the Hb tetramers (involving this 1β_2_-Glu/Val and the 2β_1_ chain, which has a hydrophobic pocket) is remarkably similar in residue-residue pair distance for both huHbS and shHbS but significantly and similarly disrupted in both huHbA and shHbB, which are not expected to polymerize in vivo. For comparison, we also performed the same analysis without standardization and observed exactly the same Pearson correlation coefficient and linear regression fitting, as expected (Fig. S1).

### Protein–solvent interactions are largely conserved between huHbA and shHbB

For several decades, it has been known that water plays a critical role in the polymerization of HbS and the absence of polymerization in HbA; it mediates both electrostatic and hydrophobic interactions^40,41,52^. This core tenet of the current understanding of the molecular mechanism of SCD helped inform our experimental design and the decision to use an explicit solvent model in our MD simulations, rather than using implicit solvent approximations, such as Poisson–Boltzmann or Generalized Born models, which are well-known to have accuracy issues for systems with many charged salt bridges or hydrogen bonds. Indeed, a recent comparison between implicit and explicit solvent simulations found poor correlations with experimental results and inherent deficiencies in models^53^. Furthermore, while the use of an implicit solvent model would have improved sampling for thermodynamic equilibrium and allowed for less time-consuming simulations, all kinetic information provided by an explicit solvent model, such as the one we used, would be lost. This limitations led Onufriev and Case^53^ to suggest using implicit solvent simulations to guide physical reasoning—a sentiment previously expressed by others^54^. It is for these reasons that we elected instead to use the more computationally expensive but accurate explicit solvent simulations in our studies.

**Figure 7:**
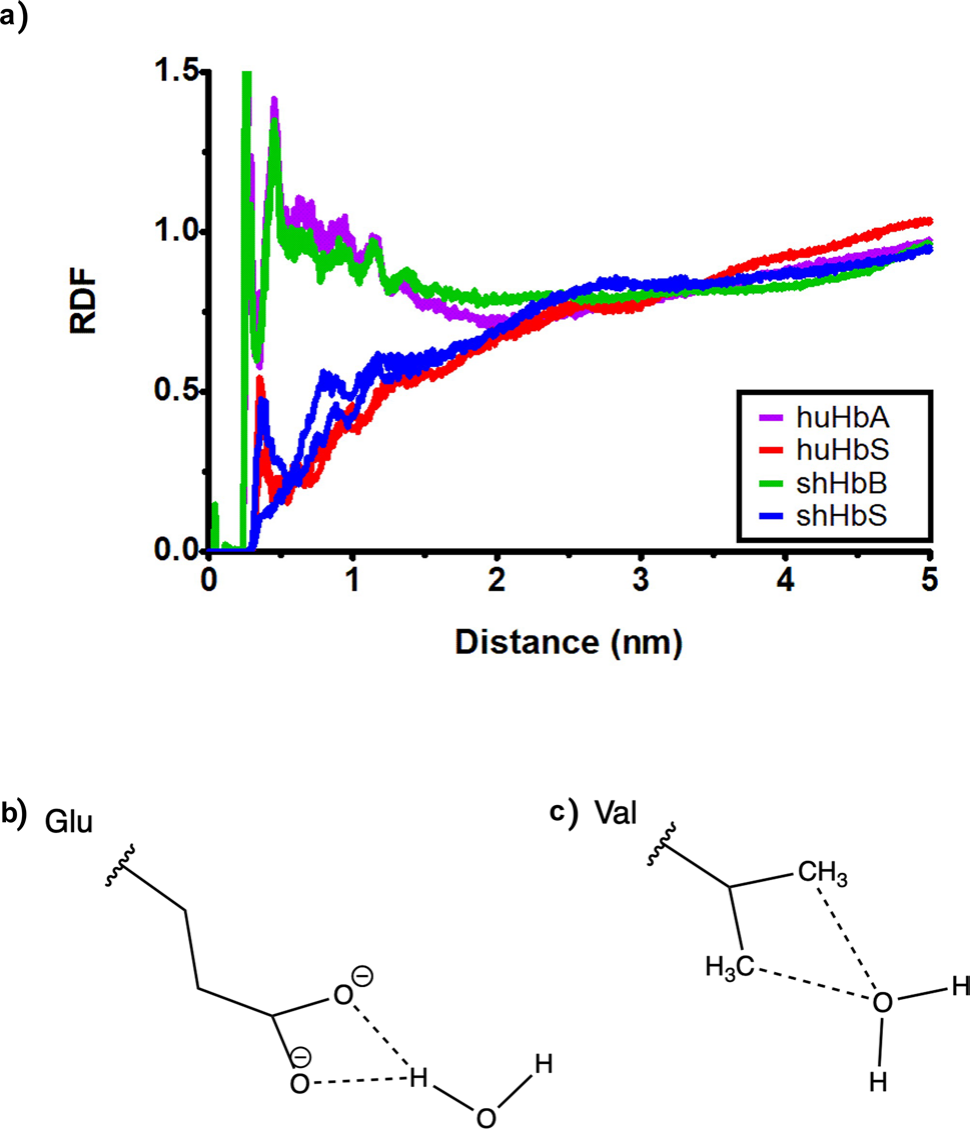
(**a**) The radial distribution functions (RDFs) between the human and sheep Hb 1ꞵ2-Glu/Val at the SCD substitution site and the solvent water oxygens, calculated as averages over each of the four 2 μs MD simulation trajectories. (**b**) The interactions between the glutamic acid side chain carboxyl group Oδ and waters. (**c**) The interactions between the valine side chain methyl groups Cɣ and waters.

Additionally, the calculations we performed with radial distribution functions to directly quantify the explicit water interactions (or lack thereof)—described below—would be impossible with implicit solvent simulations, which can only provide overall energies. An additional advantage of an explicit solvent model is that one can calculate the potential energies of an implicit solvent model, using the trajectory of an *ex*plicit solvent model. We performed these calculations for the last 1 ns of each simulation trajectory (Fig. S2). Our results for these calculations should be taken with a large grain of salt due to the number of significant approximations that have been made. Overall, we observed that the potential energies of the human variants were similar, and the sheep variants were similar, with the sheep Hb dimers about 4000 kcal/mol lower in potential energies than their human counterparts. This was not unexpected due to the numerous amino acid sequence differences between the two proteins. Notably, for both human and sheep, the sickled versions were slightly higher in potential energy—at about 500–1000 kcal/mol—than their normal counterparts throughout the trajectories. To compare the (de)hydration of critical residues at the interaction interface of the two Hb tetramers between huHbS and shHbS, we calculated the radial distribution functions (RDFs) between water and the atoms most likely to form hydrogen bonds in those residues, for all four simulation trajectories. At the SCD mutation site located at the interaction interface between the two tetramers, the RDFs between sheep 1β2-Glu5/Val5 and water are highly similar to the corresponding RDFs for human 1β2-Glu6/Val6 and water (Fig. 7). Specifically, huHbS and shHbS, which both have a mutant, hydrophobic valine in close proximity to the hydrophobic pocket (Fig. 1a,b), exhibit no significant interaction with water molecules (Fig. 7). On the other hand, both huHbA and shHbB have an acidic glutamic acid that participates in significant water interactions in the first hydration shell with its charged sidechain (Fig. 7), since it has moved significantly away from the hydrophobic pocket (Fig. 1a,b). As such, we observe in silico dehydrated valine in both huHbS and shHbS and hydrated glutamic acid in both huHbA and shHbB.

**Figure 8:**
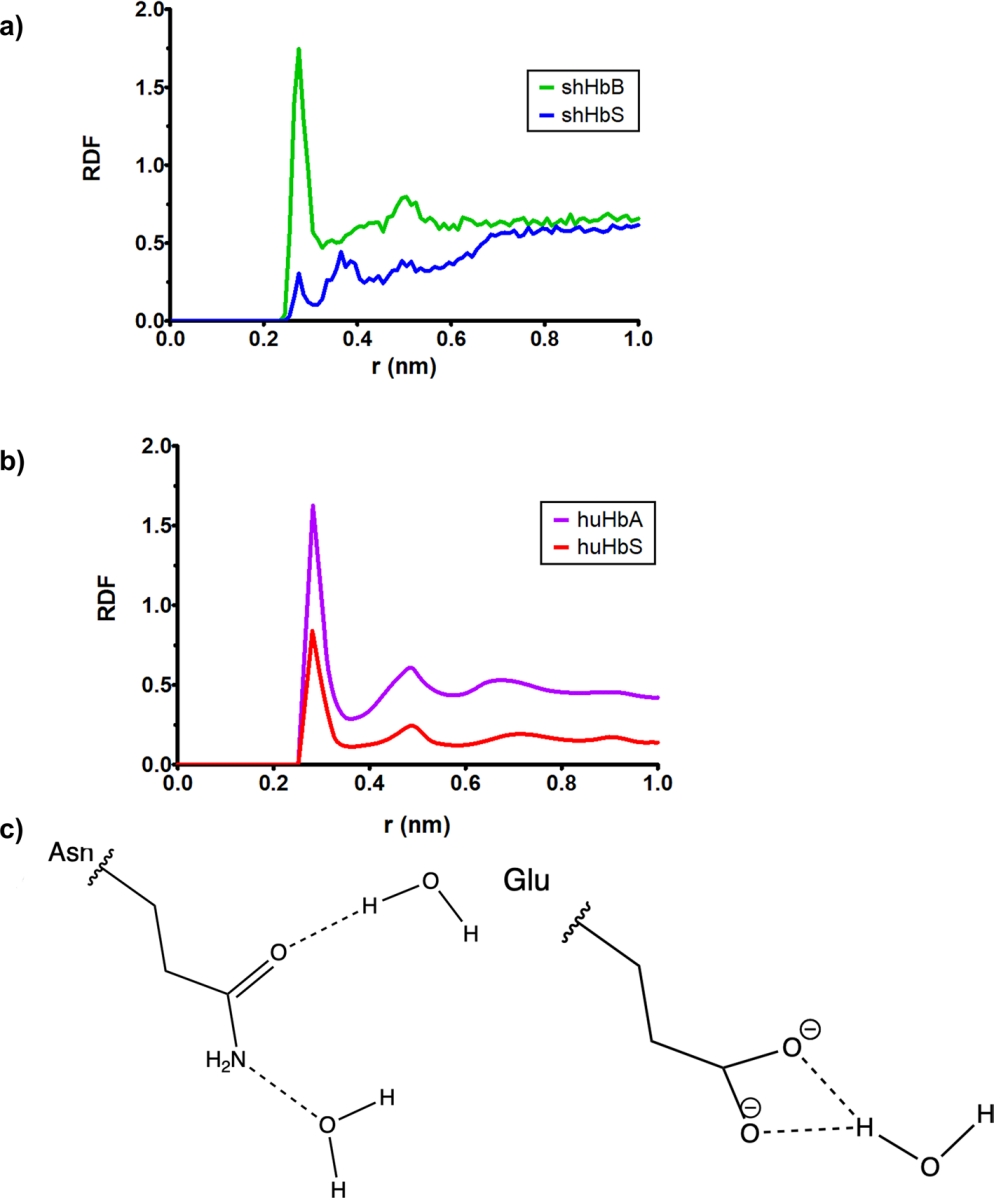
(**a**) The radial distribution functions (RDFs) between shHbB and shHbS hydrophobic pocket ꞵ1-Asn72 (corresponding to ꞵ1-Asp73 in human Hb) sidechain atom Oδ1, and the solvent water oxygens, calculated as averages over each of the 2 μs MD simulation trajectories. (**b**) Data from Galamba, 2019, demonstrating highly similar results to our data seen in Fig. 7a, with huHbA in purple, and huHbS in red. (**c**) The interactions between the asparagine side chain carboxamide group Oε and Nε and waters.

Recently, in a series of MD simulation studies of huHbA and huHbS by Galamba and Pipolo, it has been suggested that β1-Asp73, which lies near the hydrophobic pocket at the interaction interface between the two human Hb tetramers, plays a critical role in the aggregation of huHbS and also the non-aggregation of huHbA^40,41^. These authors show in their 800 ns MD simulations—using a united atom force field (GROMOS 54A7)—that the presence of β2-Glu6 in huHbA forms salt bridges and water bridged interactions in the first hydration shell that are absent in huHbS. In our longer 2 μs MD simulation of huHbA and huHbS, we observe very similar results (Fig. 8). We extended our analyses to shHbB and shHbS. However, in both sheep variants, there exists an β1-Asn72 at that equivalent position. While β1-Asp73 in huHbA and huHbS contains a carboxyl side chain which is a potential hydrogen bond acceptor, the β1-Asn72 contains a carboxamide sidechain comprised of a keto group that is a potential hydrogen bond acceptor and an amino group that is a potential hydrogen bond donor (Fig. 8b). Interestingly, we observe the same behavior such that the β1-Asn72 participates in significant solvent interactions in the first solvation for shHbB but not in shHbS.

## Discussion

To evaluate sheep as a possible animal model for the study of sickle cell disease—a molecular disease resulting from a single β-Glu6Val amino acid substitution in hemoglobin (Hb) in humans—we performed 2 μs MD simulations on the normal human and sheep variants of Hb (huHbA and shHbB) and the sickle forms (huHbS and shHbS), the longest MD simulations to date of any hemoglobin, to our knowledge. Using the high-resolution X-ray crystallographic structure of two deoxy-huHbS tetramers (PDB ID: 2HBS) (Fig. 1a), widely thought to be the starting point for subsequent aggregation and disease, we modeled shHbS based on its sequence using SWISS-MODEL, as well as their normal counterparts, huHbA and shHbB, by trivially introducing a single amino acid substitution back to its original β-Glu6, as further described in “Methods” section. The overall goal of our study was to assess whether: (1) MD simulations are able to differentiate between the normal and sickle Hb despite only a single amino acid substitution in a manner that is consistent with disease, and (2) the human and sheep variants behave similarly enough to support the development of a sheep model for the study of SCD.

Even though huHbA and huHbS differ by only a single amino acid substitution in two of the four subunits (β-Glu6Val), we observed markedly different RMSDs over the course of the 2 μs MD simulations; the RMSD of huHbA is much higher than that of huHbS, which is in line with previous experiments that demonstrate that only huHbS polymerizes and leads to subsequent SCD, while huHbA does not. Remarkably, even though the sheep hemoglobins share only 86% sequence identity with those of human, and thus 43 amino acid differences and 2 deletions, we observe remarkably similar behavior between huHbA and shHbB and between huHbS and shHbS. Specifically, the RMSD of both huHbS and shHbS levels off to about 7–10 Å after 1 μs and the RMSD of huHbA and shHbB increases to 13–15 Å, suggesting that only the huHbS and shHbS prefer to stay in the dimer formation, in -line with experiments.

We then inspected the interface between the two Hb molecules in each simulation by calculating the center of mass distances between human 1β2-Glu6/Val6 or sheep 1β2-Glu5/Val5 and their respective hydrophobic pockets on chain 2β_1_ at the interaction interface between the two tetramers (as depicted in Fig. 1a inset). We observe nearly identical results for huHbS and shHbS, while also observing markedly different results for huHbA and shHbB (Fig. 5). To more broadly explore the interactions between Glu/Val and the 2β1 chain on the adjacent Hb tetramer, we calculated the residue-residue pair center of mass distances between the Glu/Val and every other residue in the 2β1 chain. The highest Pearson correlation coefficient and linear regression fitting was observed between huHbS and shHbS (Fig. 6f), while all other comparisons had significantly lower values (Fig. 6a–e).

We also explored the water interactions with residues that are thought to be critical for Hb aggregation in huHbS and non-aggregation in huHbA using radial distribution functions (RDFs). We observe RDFs consistent with the hydration of β2-Glu6 in huHbA and also of β2-Glu5 in shHbB (Fig. 7a). On the other hand, our data indicate that the β2-Val6 in huHbS and β2-Val5 in shHbS are dehydrated, since they are interacting with their respective hydrophobic pockets (Fig. 7b). We further followed up on a recent study by Galamba that suggested that β1-Asp73 (near the hydrophobic pocket at the interaction interface between the two human Hb tetramers) plays a critical role in the aggregation of huHbS and also the non-aggregation of huHbA^40^. In the RDFs from our MD simulations, we also observed that β1-Asp73 is hydrated in huHbS but dehydrated in huHbA, matching these prior results (Fig. 8a). For shHbB and shHbS, there exists a β1-Asn72 at that equivalent position. However, it is hydrated in shHbS, but dehydrated in shHbB (Fig. 8a), suggesting that the role of the residue has been conserved, even if the residue has not.

Taken together, our MD simulation data demonstrate a marked difference between huHbA and huHbS consistent with the aggregation of huHbS and the non-aggregation of huHbA. Further, these data consistently support a strong structural and functional similarity between huHbS and shHbS, as well as huHbA and shHbB, at the molecular level. However, it should be noted that further studies on the thermodynamics of these systems—such as calculations of tetramer-tetramer interaction free energy and tetramer solubility—are required to gain a more comprehensive understanding of the behavior of shHbS in silico and the degree to which it recapitulates that of huHbS. While further studies are required to directly address the thermodynamic equilibrium or associated parameters for each system, the requisite free energy calculations using MD simulations with constraints along reaction coordinates have not yet been performed even for the dimeric human huHbA or huHbS to our knowledge.

While the studies described herein are of significance based on the timescale of our simulations because they represent a first in the field and could thus shed new light on novel aspects of Hb biology/physiology, the significance and impact of our studies are far broader than the simulations performed. By designing and performing these simulations, we have demonstrated, for the first time, that introducing the SCD-causing mutation into the sheep HbB locus will create the propensity for polymerization of the beta Hb subunits, just as is seen in human patients with SCD, the very polymerization that is responsible for sickling of the RBCs.

Overall, the data from all-atom, explicit solvent 2 µs MD simulations of huHbA, huHbS, shHbB, and shHbS strongly suggest that the domestic sheep (*Ovis aries*) represents a highly promising system in which to develop a large animal model to gain insights into the pathophysiology of SCD and to develop and test novel curative treatments for this debilitating, globally impactful disease. Indeed, validation of the physiological relevance of introducing the SCD mutation in the sheep beta globin locus led us to commence creation of a sheep model of SCD, since a point mutation in the β-globin gene that results in the replacement of the glutamic acid at position 6 of the protein with valine has never been reported to occur naturally in sheep. SCD sheep were therefore created by CRISPR/Cas9-mediated introduction of the human SCD-causing mutation into the sheep beta globin locus of fibroblasts, followed by somatic cell nuclear transfer-mediated cloning and subsequent embryo transfers. Initial analyses show the clinical presentation of these sheep mirrors that of human SCD patients^55^, thus validating the MDS modeling of the current paper. The modeling described herein, thus directly paved the way for the creation of an invaluable preclinical resource for understanding the pathophysiology of SCD and for developing and testing novel approaches to treatment.

Looking beyond the value of the sheep model of SCD that resulted from the present modeling studies, the ability of the model developed to accurately predict changes in the complex behavior of a novel protein (sheep HbS), simply by substituting a single amino acid, underscores the power of the modeling system developed and highlights its potential value in developing and testing novel treatments to prevent or reverse sickling. Given the increasing prevalence of SCD and the fact that there are currently no models that enable such predictions/testing to be performed in silico, the value of this modeling system cannot be overstated. In an even broader context, the success at predicting the behavior of tetrameric Hb suggests this model could be adapted to probe other complicated protein/biochemical processes and gain a better understanding of the mechanisms that drive their function under normal conditions and their dysfunction in disease states.

## Methods

### Experimental design

To perform MD simulations of any biological system, one must start with a high-resolution structure, typically resolved from X-ray crystallography or NMR. To date, out of the four starting structures used in our simulations, there exists in the dimeric form only a huHbS X-ray crystallographic structure (PDB ID: 2HBS), where two deoxygenated (T-state) huHbS tetramers form an extensive lateral interface to form a dimer that is thought to be the building block of intracellular sickle cell fibers. However, since shHbS is not known to occur in nature and therefore there exists no high-resolution structure, we employed SWISS-MODEL^56^ to generate a protein homology model of two shHbS tetramers in close proximity. This was accomplished using the aforementioned experimentally determined 2HBS crystal structure and the amino acid sequences for the α- and β-subunits of shHbB, with the aligned Glu → Val mutation added to the 5th position of the β-subunit sequence. As a control, we trivially modeled huHbA and shHbB by using their respective normal sequences with the same methodology as described for the model of shHbS.

The structural quality estimation for both the homology models of shHbB and shHbS (generated with SWISS-MODEL and manual alignment of the carbon backbone) indicated biophysically reasonable, high-quality structures, as summarized in Table 1, with Ramachandran plots shown in Fig. S3a–d. The QMEAN scores estimate the quality of a 3D protein structure via statistical potentials of mean force, where a score of 0 is the maximum value, and a score greater than − 4.00 is considered the cutoff for a good-quality model^57,58^. For our modeled structures of shHbB and shHbS, the QMEAN scores were − 1.30 and − 1.36, respectively, well-above the cutoff. In addition, the Ramachandran plots of both modeled structures had 99.25% of Φ and Ψ angles determined to be Ramachandran favored, with 0.00% being outliers^59^. For reference, conducting the same analyses on the experimentally determined huHbS crystal structure directly from the PDB (ID: 2HBS) gave a QMEAN score of − 0.57, and 98.50% of Φ and Ψ angles being Ramachandran favored, with 0.09% being outliers. When we modeled the normal huHbA sequence (substituting back β-Glu6), the QMEAN score was − 0.81, 99.35% of the Φ and Ψ angles were Ramachandran favored, and 0.00% were outliers. Taken together, these results suggest that all of our homology models are high-quality and comparable to experimentally derived structures according to these measures.

**Table 1:** SWISS-MODEL structural statistics for Hb structures.

	QMEAN	Ramachandran favored%	Outlier%
huHbA	− 0.81	99.35	0.00
huHbS (using 2HBS crystal structure)	− 0.57	98.50	0.09
shHbB	− 1.30	99.25	0.00
shHbS	− 1.36	99.25	0.00

All-atom, explicit solvent MD simulations were conducted using Wake Forest University’s DEAC High-Performance Computing Cluster^60^. Histidine protonation states for the starting structures were specified to mirror those previously used in multiple MD simulations of deoxy-Hb^51–62^. The software programs VMD 1.9.4^63^ and NAMD 2.14^64^ were used to set up and solvate the simulations using the TIP3P water model^65^ at a sodium chloride concentration of 154 mM—the intracellular NaCl concentration in RBCs. Using explicit water molecules resulted in very large sizes of our systems, with the shHbS system having 18,164 atoms before solvation with 36,074 water molecules and an additional 214 sodium and chloride ions, giving a total of 126,600 atoms for that system, with very similar large sizes for the other systems used. NAMD and the CHARMM36 force fields^66,67^ were used to carry out the simulations at room temperature (298 K) using the well-studied constant temperature and pressure (i.e., NPT) Langevin Dynamics ensemble^68^.

The programming language R 4.0.2^69^ and the package Bio3D 2.4-1^70^ were used for certain simulation trajectory analyses, such as root mean square deviation (RMSD), root mean square fluctuation (RMSF), and dynamic cross-correlated motion analysis. Such analyses were conducted to respectively inform us if the overall structure of the protein/two tetramers was indicative of significant structural rearrangement, if certain amino acids or segments of the protein appeared to exhibit greater flexibility, if certain amino acids or segments of the protein appeared to be moving in a coordinated fashion, suggesting interaction between different parts of the protein or two tetramers.

VMD was used for additional analyses, including the radial distribution function (RDF) of water—informing us if hydrogen bonds appeared to be forming, suggesting solvent exposure—and center of mass distances. The center of mass distances between the 1β2-Val6/Glu6 (human) or 1β2-Val5/Glu5 (sheep) and every other residue in the 2β1 chain, were calculated over the last 1 μs of the simulations—after the RMSDs for huHbS and shHbS had stabilized—at intervals of 50 ns. Subsequently, each dataset was standardized individually. After standardization, center of mass distances from the four trajectories (n = 2900 each) were all compared pairwise to determine the strength of a linear relationship between the center of mass distances (between Val/Glu and every residue in the 2β1 chain) of two trajectories. Since human and sheep Hb have different β-chain lengths, we aligned the sequences and assigned each residue an index number to be able to directly compare analogous residues. The center of mass distance values were then paired to their corresponding index number for each trajectory. Following index alignment and standardization via the equation $z=\frac{x-\mu }{\sigma }$ (where x is the raw data value, μ is the mean, and σ is the standard deviation), each trajectory was compared to another by the calculation of a Pearson’s correlation coefficient for each pair.

### Supplementary Information

Supplementary Figures.

## Data Availability

The main data supporting the findings of this study are available within the article and its Supplementary Information. The X-ray structures used in this study is available in the Protein Data Bank database under accession code 2HBS. Additional data are available from the corresponding author SSC (choss@wfu.edu) upon reasonable request.
