# Supplementary material for: Evaluating sheep hemoglobins with MD simulations as an animal model for sickle cell disease
Source: Sci Rep. 2024 Jan 2;14:276. doi: 10.1038/s41598-023-50707-y (PMC10761887; doi:10.1038/s41598-023-50707-y)
Supplement: Supplementary file 1 — Supplementary Figures. [file 41598_2023_50707_MOESM1_ESM.pdf]

Supplementary Information for:

Evaluating Sheep Hemoglobins with MD Simulations as an Animal Model for Sickle Cell Disease

Caroline E. Kuczynski, Christopher D. Porada, Anthony Atala, Samuel S. Cho, and Graça Almeida-Porada

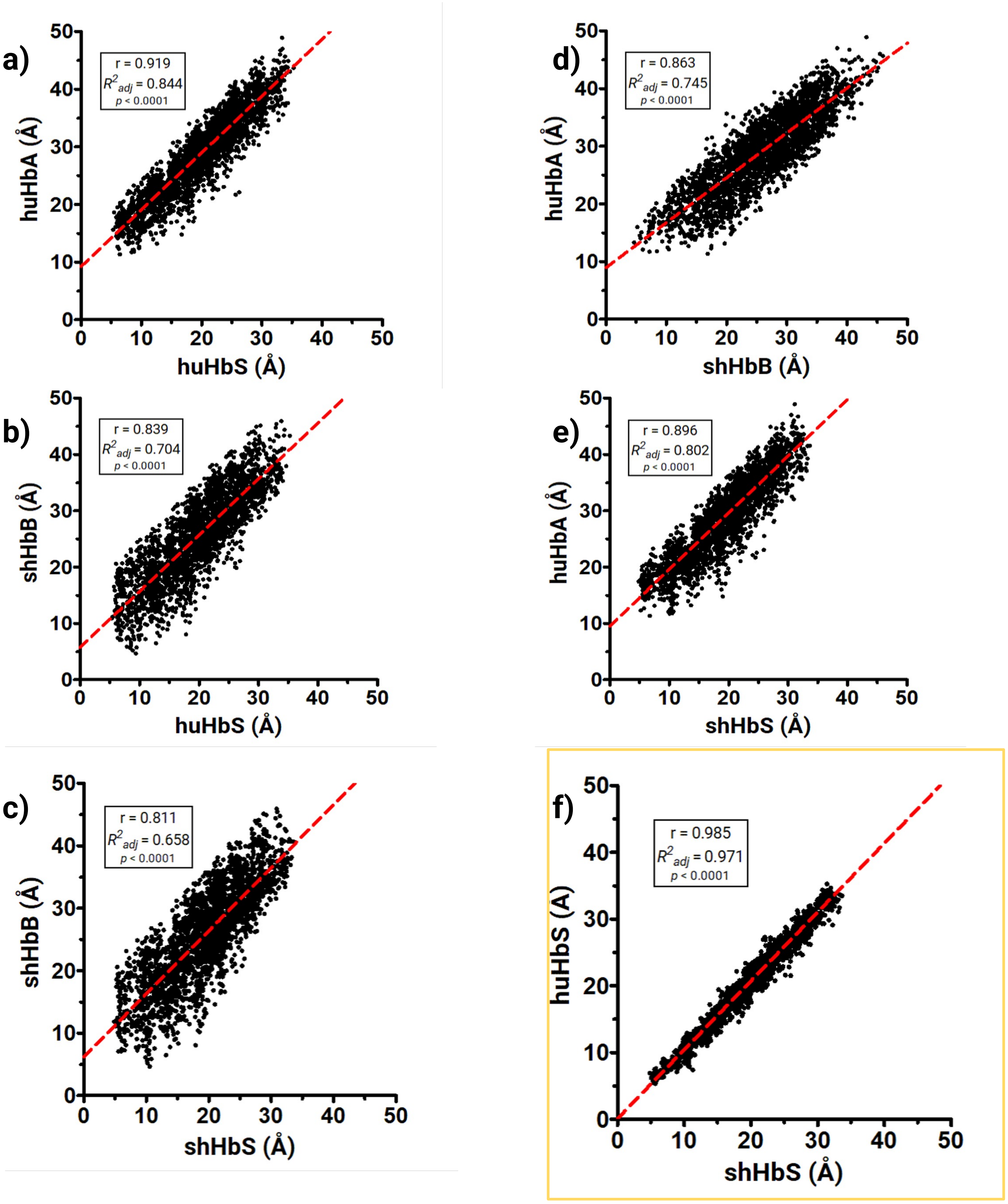
a)
r = 0.919
R^2_{adj} = 0.844
p < 0.0001
huHbA (Å)
huHbS (Å)
d)
r = 0.863
R^2_{adj} = 0.745
p < 0.0001
huHbA (Å)
shHbB (Å)
b)
r = 0.839
R^2_{adj} = 0.704
p < 0.0001
shHbB (Å)
huHbS (Å)
e)
r = 0.896
R^2_{adj} = 0.802
p < 0.0001
huHbA (Å)
shHbS (Å)
c)
r = 0.811
R^2_{adj} = 0.658
p < 0.0001
shHbB (Å)
shHbS (Å)
f)
r = 0.985
R^2_{adj} = 0.971
p < 0.0001
huHbS (Å)
shHbS (Å)

Figure S1. Correlation between center of mass distances between human 1β_2-Val/Glu6 or sheep 1β_2-Val/Glu5 and all other residues in chain 2β_1, calculated from 1-2 μs, at every 50 ns, before standardization. Shown are the correlations between (a) huHbA and huHbS, (b) shHbB and huHbS, (c) shHbB and shHbS, (d) huHbA and shHbB, (e) huHbA and shHbS, and (f) huHbS and shHbS, each with an inset consisting of the Pearson correlation coefficient (r), linear regression fitting (R^2) and p-value (p).

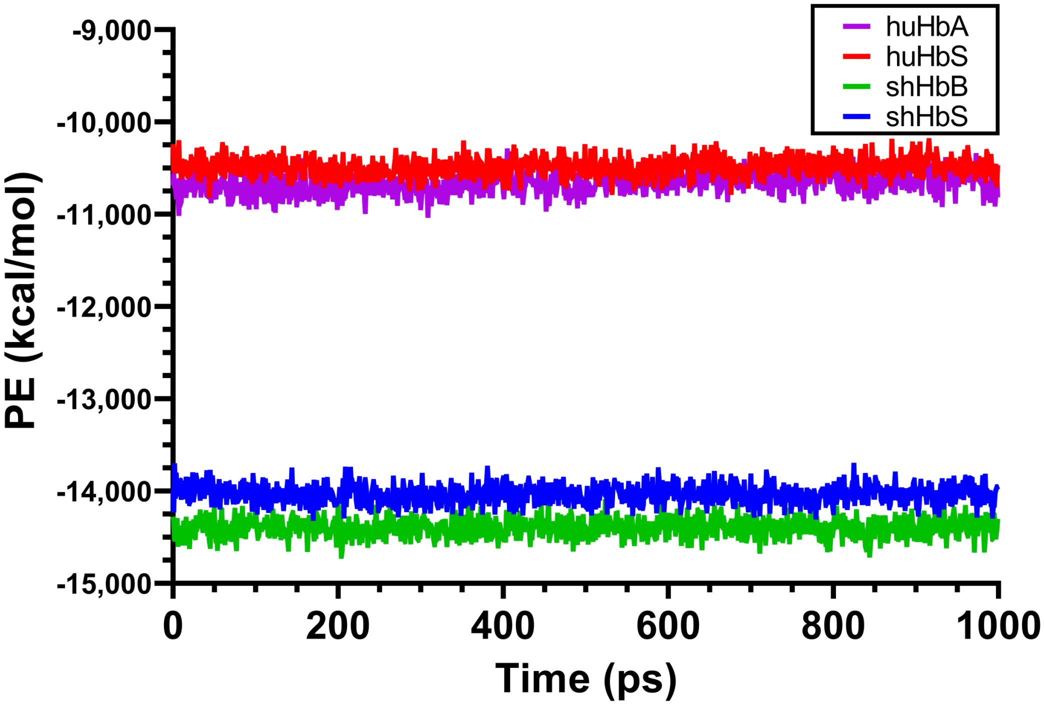

huHbA
huHbS
shHbB
shHbS
PE (kcal/mol)
-9,000
-10,000
-11,000
-12,000
-13,000
-14,000
-15,000
0
200
400
600
800
1000
Time (ps)

Figure S2. Potential energies of an implicit solvent model, calculated using the last nanosecond of each 2 μs trajectory conducted using an explicit solvent model, for huHbA, huHbS, shHbB, and shHbS.

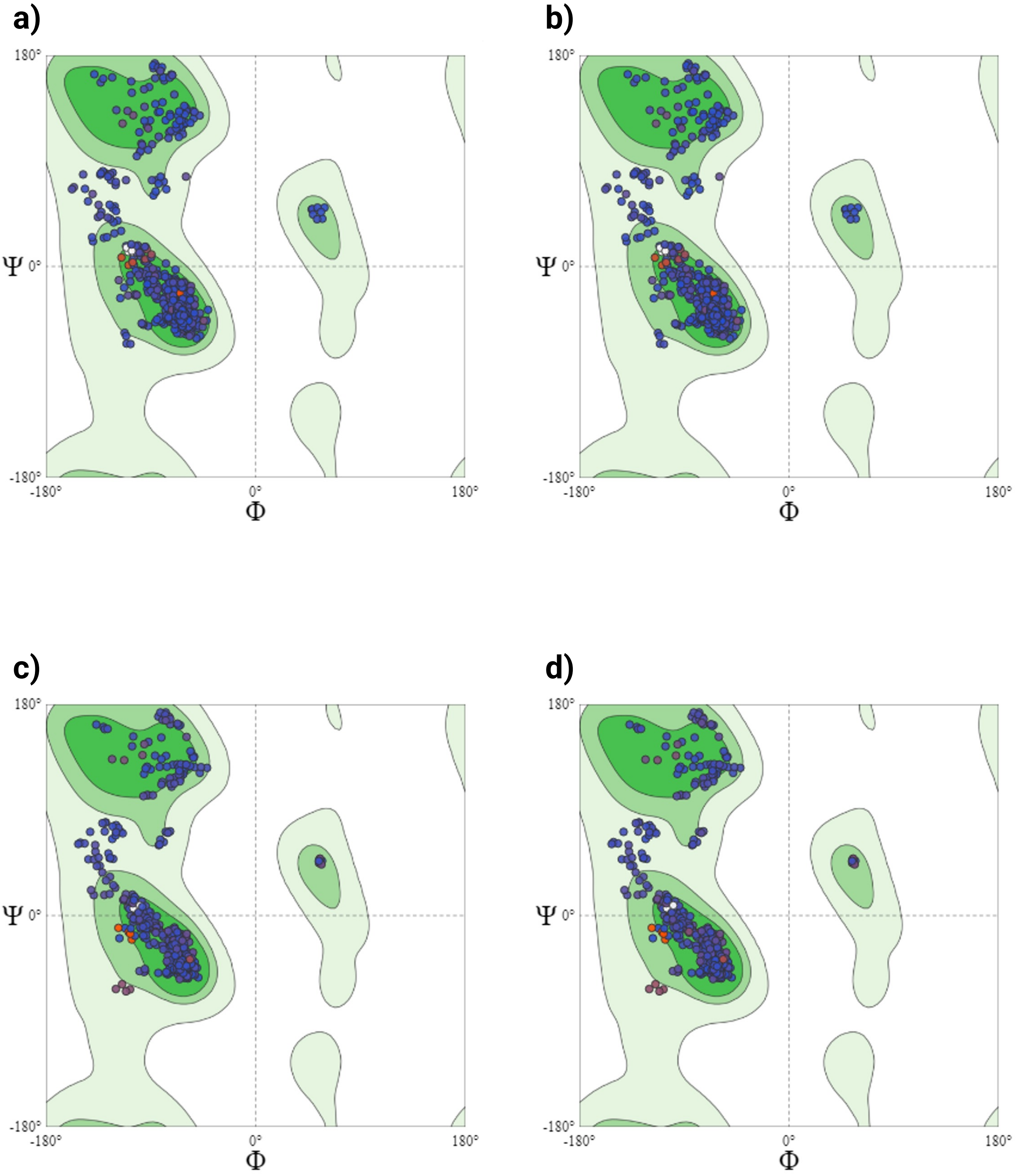

a)
b)
c)
d)
180°
Ψ 0°
-180°
-180°
0°
180°
Φ

Figure S3. Ramachandran plots, generated using the SWISS-MODEL Structure Assessment tool, of starting structures used in MD simulations after setup with NAMD for a) huHbA, b) huHbS, c) shHbB, and d) shHbS.
